# Supplementary material for: A non-conducting role of the Cav1.4 Ca2+ channel drives homeostatic plasticity at the cone photoreceptor synapse
Source: eLife. 2024 Nov 12;13:RP94908. doi: 10.7554/eLife.94908 (PMC11556788; doi:10.7554/eLife.94908)
Supplement: Supplementary file 1. [file elife-94908-supp1.pdf]

Supplementary Table 1. Statistical analysis of visible platform swim test disaggregated by sex

Male versus Female WT (Photopic)	SS	DF	MS	F (DFn, DFd)	P value
Trial x Genotype	1125	5	224.9	F (5, 45) = 0.6060	P=0.6956
Trial	3564	5	712.9	F (5, 45) = 1.921	P=0.1096
Genotype	273.6	1	273.6	F (1, 9) = 0.6616	P=0.4370
Subject	3722	9	413.5	F (9, 45) = 1.114	P=0.3727
Residual	16703	45	371.2		
**Male versus Female G369i KI (Photopic)**	SS	DF	MS	F (DFn, DFd)	P value
Trial x Genotype	1457	5	291.4	F (5, 45) = 0.5362	P=0.7477
Trial	3516	5	703.2	F (5, 45) = 1.294	P=0.2833
Genotype	1150	1	1150	F (1, 9) = 0.8168	P=0.3897
Subject	12675	9	1408	F (9, 45) = 2.592	P=0.0168
Residual	24451	45	543.4		
**Male versus Female Cav1.4 KO (Photopic)**	SS	DF	MS	F (DFn, DFd)	P value
Trial x Genotype	1736	5	347.1	F (5, 35) = 0.6597	P=0.6563
Trial	993	5	198.6	F (3.056, 21.39) = 0.3775	P=0.7737
Genotype	78.37	1	78.37	F (1, 7) = 0.03788	P=0.8512
Subject	14481	7	2069	F (7, 35) = 3.932	P=0.0029
Residual	18416	35	526.2		
**Male versus Female WT (Scotopic)**	SS	DF	MS	F (DFn, DFd)	P value
Trial x Genotype	862	5	172.4	F (5, 45) = 0.6907	P=0.6331
Trial	914.5	5	182.9	F (5, 45) = 0.7328	P=0.6027
Genotype	634.7	1	634.7	F (1, 9) = 2.121	P=0.1793
Subject	2693	9	299.3	F (9, 45) = 1.199	P=0.3193
Residual	11232	45	249.6		
**Male versus Female G369i KI (Scotopic)**	SS	DF	MS	F (DFn, DFd)	P value
Trial x Genotype	7648	5	1530	F (5, 45) = 2.581	P=0.0390
Trial	1644	5	328.8	F (5, 45) = 0.5547	P=0.7339
Genotype	4712	1	4712	F (1, 9) = 4.716	P=0.0580
Subject	8993	9	999.2	F (9, 45) = 1.686	P=0.1206
Residual	26671	45	592.7		
**Male versus Female Cav1.4 KO (Scotopic)**	SS	DF	MS	F (DFn, DFd)	P value
Trial x Genotype	1727	5	345.4	F (5, 35) = 1.470	P=0.2246
Trial	12215	5	2443	F (2.802, 19.62) = 10.40	P=0.0003
Genotype	96.33	1	96.33	F (1, 7) = 0.1346	P=0.7246
Subject	5011	7	715.9	F (7, 35) = 3.046	P=0.0131
Residual	8225	35	235		

Data were analyzed by two-way repeated measures ANOVA. SS, sums of squares; DF, degrees of freedom; MS, mean square; F(DFn, DFd), F statistic.
